# Assessment of the Sensitizing Potential of Processed Peanut Proteins in Brown Norway Rats: Roasting Does Not Enhance Allergenicity

**DOI:** 10.1371/journal.pone.0096475

**Published:** 2014-05-07

**Authors:** Stine Kroghsbo, Neil M. Rigby, Philip E. Johnson, Karine Adel-Patient, Katrine L. Bøgh, Louise J. Salt, E. N. Clare Mills, Charlotte B. Madsen

**Affiliations:** 1 National Food Institute, Technical University of Denmark, Søborg, Denmark; 2 Institute of Food Research, Norwich Research Park, Colney, Norwich, United Kingdom; 3 INRA, UR496, Unité d’Immuno-Allergie Alimentaire, Jouy-en-Josas, France; University of Kansas Medical Center, United States of America

## Abstract

**Background:**

IgE-binding of process-modified foods or proteins is the most common method for examination of how food processing affects allergenicity of food allergens. How processing affects sensitization capacity is generally studied by administration of purified food proteins or food extracts and not allergens present in their natural food matrix.

**Objectives:**

The aim was to investigate if thermal processing increases sensitization potential of whole peanuts via the oral route. In parallel, the effect of heating on sensitization potential of the major peanut allergen Ara h 1 was assessed via the intraperitoneal route.

**Methods:**

Sensitization potential of processed peanut products and Ara h 1 was examined in Brown Norway (BN) rats by oral administration of blanched or oil-roasted peanuts or peanut butter or by intraperitoneal immunization of purified native (N-), heated (H-) or heat glycated (G-)Ara h 1. Levels of specific IgG and IgE were determined by ELISA and IgE functionality was examined by rat basophilic leukemia (RBL) cell assay.

**Results:**

In rats dosed orally, roasted peanuts induced significant higher levels of specific IgE to NAra h 1 and 2 than blanched peanuts or peanut butter but with the lowest level of RBL degranulation. However, extract from roasted peanuts was found to be a superior elicitor of RBL degranulation. Process-modified Ara h 1 had similar sensitizing capacity as NAra h 1 but specific IgE reacted more readily with process-modified Ara h 1 than with native.

**Conclusions:**

Peanut products induce functional specific IgE when dosed orally to BN rats. Roasted peanuts do not have a higher sensitizing capacity than blanched peanuts. In spite of this, extract from roasted peanuts is a superior elicitor of RBL cell degranulation irrespectively of the peanut product used for sensitization. The results also suggest that new epitopes are formed or disclosed by heating Ara h 1 without glucose.

## Introduction

Food allergy is an adverse reaction to an otherwise harmless food or food component that involves an abnormal response of the immune system to specific proteins in foods. It is an allergen-specific immunologic response mediated by IgE. One of the major unanswered questions in food allergy research is what makes some foods and food proteins more allergenic than others. Seeking such answer is difficult since the proteins involved in sensitizing or eliciting allergic reactions may have undergone extensive modifications during food processing or be presented within complex food matrices. Certainly, both food processing and structure of the food matrix may impact allergenicity of food allergens [1]–[4].

Food processing may involve many different and complex physicochemical changes of the food which make it difficult to study and predict how processing affects the allergenic potential of a food protein. Moreover, alterations induced by processing may change the way in which a food protein is digested, influence allergen release from the food matrix or affect the form in which it is taken up across the epithelial barrier and presented to the immune system. Hence, the impact of processing on allergenicity of a food protein may be different from food to food or protein to protein. It is important to notice that the majority of proteins within foods may become insoluble after food processing. By this way, only a small part of proteins in processed foods are examined for changes in allergenicity by most serological and clinical analyses, as they are usually performed with food proteins extracted by simple salt solutions [2], [5]–[7]. Different processing methods may impact the allergenic potential of foods or proteins, but there is no general rule on how different allergenic foods or proteins respond to physical, chemical or biochemical exposures during processing [7]. Allergenicity in the terms of IgE-binding may be decreased, unaltered or increased [2], [6]–[8] and may be influenced by food processing conditions, variability in the allergen composition of the whole food, food matrix structure, multiplicity and types of IgE epitopes, thermodynamics of the allergen, and stability of the scaffold [7], [8]. The most common types of modifications that food proteins undergo during processing include protein unfolding and aggregation, in addition to chemical modification, thus both the secondary and tertiary structure of native proteins can be altered as a consequence of processing [2], [9].

Thermal processing is one of the most commonly used methods in food processing and depending on the time and temperature, thermal processing may alter protein structure and thereby the allergenicity of food proteins [8]. One of the most important chemical modifications occurring in foods during thermal processing is the reaction between free amino groups (generally lysine residues) of proteins and the aldehyde and ketone groups of sugars known as the Maillard reaction (non-enzymatic browning). This complex reaction occurs during heating of proteins leading to formation of a variety of poorly characterized molecules responsible for different odors and flavors [10]. The extent of glycation depends on different factors such as heating temperature and duration, and the concentration of reducing sugars [10], [11]. The impact of Maillard reaction on IgE-binding has been studied for food allergens from milk [10]; peanut [11]–[17]; buckwheat [18]; scallop [19]; squid [20]; cherry [21]; apple [22]; and hazelnut [23]. Results from these studies are ambiguous and show that glycation can both increase and decrease IgE-binding. In spite of these studies it has not been possible to set up a general rule on how non-enzymatic glycation affects allergenicity of food proteins.

Peanuts are easy and cheap to produce and are consumed worldwide and one of the most widely processed food products in the western world. The majority of peanuts consumed in westernized countries have been processed by roasting whereas boiling is the preferred processing method in Asia and Africa [24]. Peanut seems to have an increased IgE-binding after dry-roasting as a whole food [11], [12] compared to cooked or fried peanut [13], [17]. However when looking at the two major peanut allergens, Ara h 1 and Ara h 2 they seem to be affected in different ways [16]. Furthermore there is no general consensus on how IgE-binding is affected by different forms of thermal processing for the two individual allergens [11], [14]–[17], [25]–[29] suggesting that the type of parameters such as allergen, food structure, and thermal processing may be of great importance when studying the impact of processing on food allergenicity. Moreover, the impact of food processing and food matrix on the sensitization capacity of peanut food allergens has only been rarely studied [30], [31].

It has been shown that roasting of peanuts increases IgE-binding. This has been interpreted as roasting increases allergenicity of peanut proteins. To investigate if roasting increases sensitization capacity of peanut proteins, blanched (mildly heated) peanut, oil-roasted peanut and peanut butter were examined in an oral Brown Norway rat model for food allergy. In addition, process-modified purified Ara h 1 was used to study the impact of heating and heat-induced glycation on sensitization potential of a well-characterized major peanut allergen.

## Materials and Methods

### Ethics Statement

Animal experiments were carried out at the DTU Food (Mørkhøj, Denmark) facilities. Ethical approval was given by the Danish Animal Experiments Inspectorate. The authorization number given: 2004/561-917. The experiments were overseen by the National Food Institutes in-house Animal Welfare Committee for animal care and use.

### Purification and Processing of Peanut Proteins

Native Ara h 1 and Ara h 2 were purified from peeled raw red shelled peanuts (obtained from a local supplier, UK). Ara h 1 was purified as described previously by Marsh *et al*. [32] and Ara h 2 was purified as described by Johnson *et al*. [33]. Concentrations of purified native and process-modified peanut proteins were determined by amino acid analysis [34].

#### Heating and glycation of native Ara h 1

Native (NAra h 1) and processed (HAra h 1, GAra h 1) peanut proteins were obtained as described in Blanc *et al*. [17]. Heating of Ara h 1 induced hydrolysis, partial loss of secondary structure and aggregation regardless of whether glucose was present. Heating alone (HAra h 1) resulted in formation of aggregates comprised of lower *M_r_* polypeptides (∼6–67 kDa) whereas aggregates of higher *M_r_* polypeptides (>200 kDa) were formed by heating in the presence of glucose (GAra h 1) [17]. Samples were kept at −70°C in aliquots until use for animal experiments and immunochemical analysis.

### Preparation of Peanut-water Mixtures for Use in Oral Sensitization Studies

Blanched and oil-roasted peanuts were kindly provided by Kraft Foods Norway (Runners peanuts grown in Argentina). Blanched peanuts were steam blanched at 120–130°C for 10–15 min to remove skin and prevent microbial growth. Oil-roasted peanuts were pretreated by blanching before oil roasting in a continuous roaster (150–160°C for 3–5 min). Peanut butter was purchased from a local retail outlet (Sunpat peanut butter, smooth; produced from roasted peanuts, 48.9% fat, 25.0% protein; UK). For oral dosing of rats by gavage, blanched or roasted peanuts or peanut butter were milled in a food processor together with water to obtain nut-water mixtures. Samples of peanut-water mixtures were stored at −20°C until use for animal experiments.

### Preparation of Peanut Product Extracts for Analysis of Antibody Response

For immunochemical analysis of rat sera, peanut protein extracts were prepared from each of the peanut-water mixtures by addition of 5 mL carbonate buffer (20 mM, pH 9.6) per 2 g of peanut-water mixture before homogenization using an Ultraturrhax (15,000–20,000 rpm; IKA Werke GmbH & Co, Staufen, Germany). Then carbonate buffer was added to obtain a final volume of 40 mL, incubated overnight under agitation at 4°C followed by centrifugation (6000×*g*, 45 min, 4°C) and collection of supernatant containing peanut protein extract. Protein concentrations in extracts were estimated by the method of bicinchoninic acid (Pierce BCA Protein Assay Kit; Thermo Fisher Scientific Inc., Rockford, IL, USA) according to manufacturer’s instructions. The yield of protein extraction was calculated to be 7.6 mg/mL (blanched peanuts), 3.6 mg/mL (roasted peanuts) and 4.4 mg/mL (peanut butter).

**Figure 1:**
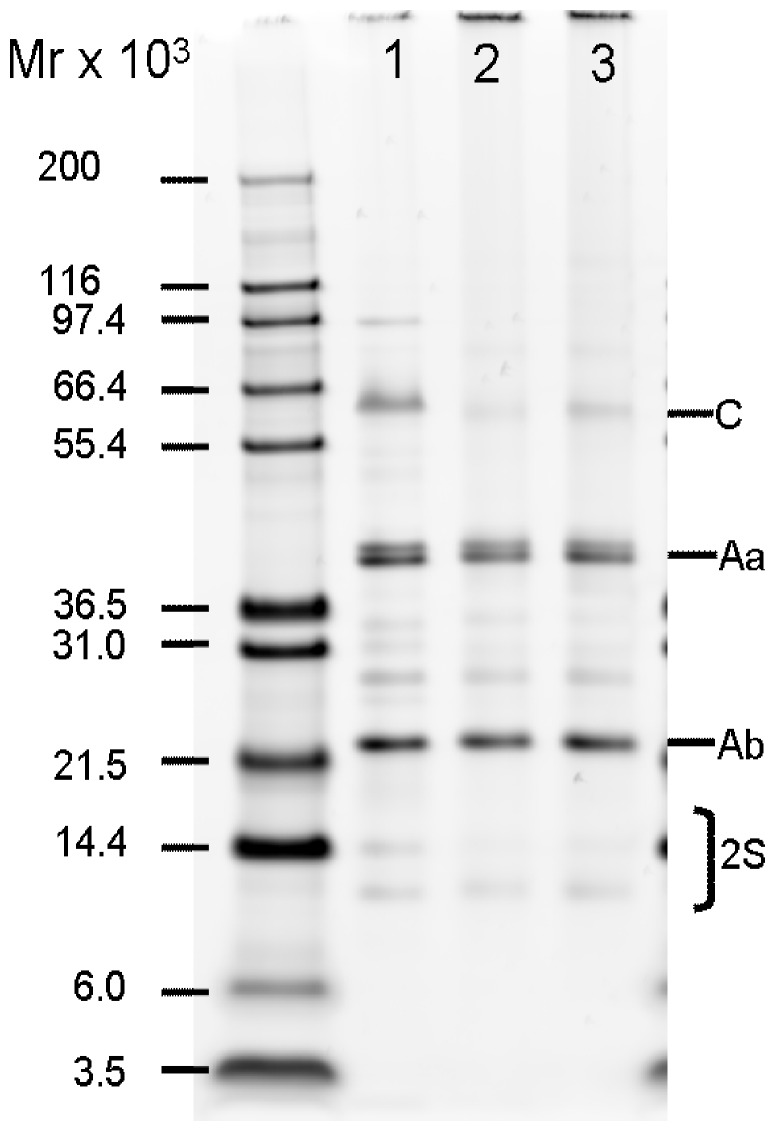
Identification of soluble peanut allergens in peanut product extracts. Extracts of blanched peanuts (lane 1), roasted peanuts (lane 2) and peanut butter (lane 3) were analyzed by SDS-PAGE under reducing conditions. Mark12 MW Standard (Invitrogen) was used as molecular mass standard and the gel was stained using a Coomassie-based stain (SimplyBlue SafeStain; Invitrogen). Calculated protein loading per lane is 0.65 µg based on determination of protein concentration by BCA assay. Protein Identification: C (Conarachin, 7S globulin), Aa (Arachin acidic subunits, 11S globulin), Ab (Arachin basic subunits, 11S globulin), 2S (2S albumins).

The presence of Ara h 1, Ara h 2 and other relevant peanut proteins in the peanut product extracts was confirmed by SDS-PAGE under reducing conditions (Figure 1). SDS-PAGE was performed using NuPAGE 4–12% Bis-Tris gels (Invitrogen, Paisley, UK) with MES running buffer according to manufacturer’s instructions. Mark12 MW Standard (Invitrogen) was used as molecular mass standards and gels were stained using a Coomassie-based stain (SimplyBlue SafeStain; Invitrogen). After destaining, gels were scanned using Pharos FX plus Imager and analyzed using Quantity One software v4.6.1 (BioRad, Hertfordshire, UK).

### Animals

Inbred high IgE-responder Brown Norway (BN) rats were from our in-house colony at the DTU Food (Denmark). Rats were weaned at three weeks of age. Rats, 4–6 weeks of age, were randomized into groups. Randomization was done to ensure that animals from the same litter were distributed as evenly as possible in different groups and that the age distribution in the groups was comparable. Animals were housed in macrolon cages (two/cage) at 22±1°C, relative humidity 55 ± 5%, air change 10 times/h, and electric light from 9.00 am – 9.00 pm. Animals had hidings and a wooden block. Diet and acidified water (pH 3.5) were given *ad libitum*. Animals were inspected twice daily and body weights recorded weekly. To avoid tolerization against peanut proteins, animals were bred for at least three generations on a diet without *leguminosa* developed and produced at the DTU Food (Denmark) [35]. Diet samples were analyzed using Peanut Assay Kit (Neogen Europe, Flintshire, UK) and Soy Residue kit (Elisa Systems, Queensland, Australia) to ensure that the diet was not contaminated with minor amounts of peanut or cross-reacting soy proteins. Assay procedure and detection limits were as described previously [35]. At termination of sensitization experiments all animals were anaesthetized by carbon dioxide inhalation and killed by exsanguinations.

### Animal Sensitization Studies

Positive control sera for native Ara h 1 and Ara h 2 were produced by intraperitoneal (i.p.) immunization with 50 µg of peanut antigen absorbed on 12 mg Al(OH)_3_ per rat at day 1 and 10 µg antigen per rat at day 21, 35 and 49. Blood was collected at sacrifice (day 56). In all experiments sera were obtained from blood samples and stored at −20°C until analysis.

Sensitization capacity of peanut products and native and processed Ara h 1 was examined in two different rat models. Peanut products were studied using an oral model (intra-gastric dosing of rats) to mimic human intake whereas an i.p. model was used for studies of native and processed Ara h 1 because of the lower amounts of purified protein required compared to the oral model.

Dosing or blood drawing of animals did not result in any adverse events.

#### Feeding study with peanut products

Groups of 16 BN rats (8 per sex) were dosed by gavage each day for 42 days with 0.5 mL per rat per day of peanut-water mixtures of blanched or roasted peanuts or peanut butter to study whether food processing influences the oral sensitization capacity of Ara h 1 in its natural matrix. Peanut-water mixtures were prepared and adjusted so rats were dosed with ∼2 mg Ara h 1 and ∼1 mg Ara h 2 per rat per day. Concentrations of Ara h 1 and Ara h 2 in peanut products were estimated based on data from ‘The official Danish Food Composition Database version 7.01’ [www.foodcomp.dk/v7; Technical University of Denmark) and personal communication [Neil M Rigby; IFR, Norwich, UK]. Dosing regime and concentration of Ara h 1 and Ara h 2 were chosen based on results obtained in a preliminary study (data not shown). A group of 16 untreated BN rats was included as control. Blood was collected under Hypnorm/Dormicum anesthesia before study initiation and one week after the last dosing.

#### I.p. study with native, heated and glycated Ara h 1

To examine whether heating and glycation affects the sensitization capacity of purified native Ara h 1, groups of 12 BN rats (6 per sex) were immunized i.p. three times (day 0, 14 and 28) with none (control) or 200 µg of NAra h 1, HAra h 1 or GAra h 1 in 0.5 mL PBS (pH 7.2) per rat per immunization. Blood was collected before study initiation and one week after the last immunization.

All dosing and handling of animals were done in the animal room by experienced animal technicians having an education approved by the Danish Animal Experiments Inspectorate.

### Enzyme-Linked ImmunoSorbent Assay (ELISA)

Measurement of specific IgG1, IgG2a and IgE against native Ara h 1 (NAra h 1) was performed as described previously [35]. For details about washing procedures, development of enzymatic reaction and calculation of titer values see Kroghsbo *et al*. [35], [36].

#### ELISAs for detection of specific IgG1 and IgG2a

For measurement of the specific IgG1 and IgG2a response plates (96-well, MaxiSorp; Nunc, Roskilde, Denmark) were coated overnight at 4°C with 0.5 µg/mL of Ara h 2 or 1.0 µg/mL of HAra h 1, GAra h 1 or peanut product extract in carbonate buffer (pH 9.6; 15 mM Na_2_CO_3_, 35 mM NaHCO_3_). For detection of specific IgG1 and IgG2a against HAra h 1 and GAra h 1, plates were blocked for 1 h at 37°C with 200 µL/well of 1% BSA in PBS/Tween buffer (PBS-T, 0.05% Tween 20). A blocking step was not performed for plates used for detection of specific IgG1 and IgG2a against Ara h 2 or peanut product extracts as optimization procedures showed no effect of blocking on background absorbance values. Then, plates were incubated with serially diluted rat sera in PBS-T for 1 h at RT before incubation with horseradish peroxidase (HRP)-labeled mouse anti-rat IgG1 or IgG2a (1∶2000; Zymed, San Francisco, CA, USA) in PBS-T for 1 h at RT followed by development with TMB-one (Kem-En-Tec, Copenhagen, Denmark) for 10 min in the dark. Detection limits were calculated to an absorbance value of 0.1 for all IgG1 assays and 0.2 for all IgG2a assays.

#### Inhibition ELISAs for examination of binding capacity of specific IgG1

Assay procedures were as described for measurement of specific IgG1 except that sera were preincubated with inhibitor solutions. Serum pools were diluted to reach an OD between 0.8 and 1.0 and preincubated (1 h at RT) with serial tenfold dilutions of NAra h 1, HAra h 1 or GAra h 1 (0.1 ng/mL–100 µg/mL) or peanut product extracts (1 ng/mL–1000 µg/mL) before triplicates of serum/inhibitor mix (and sera with no inhibitor as a control) were added to the wells. Results were expressed as *B*/*B_0_* where *B* corresponds to the specific IgG1-binding to immobilized protein when a known concentration of inhibitor is present and *B_0_* corresponds to the binding in the absence of inhibitor. For each serum pool the concentration of inhibitor that inhibits 50% of the binding to the coated antigen/extract (IC_50_) was determined, where an increase in IC_50_ value is correlated to a lower IgG reactivity of the product used as inhibitor. Analysis of inhibition curves by GraphPad Prism (GraphPad Software, San Diego, CA, USA) showed that inhibition curves were parallel (slopes were not significantly different) which is important for appropriately comparison of IC_50_ values.

#### Antibody-capture ELISAs for detection of specific IgE

To avoid the interference of the much higher level of IgG than IgE, assays based on selective IgE capture was established for detection of antigen-specific IgE responses. Specific IgE against Ara h 2 or processed Ara h 1 was measured by coating plates overnight at 4°C with 0.5 µg/mL of mouse anti-rat IgE (HPMAB-123 HybriDomus, Cytotech, Hellebæk, Denmark) in carbonate buffer. After blocking of remaining active sites overnight at 4°C with PBS-T containing 1% rat serum from naïve rats, plates were incubated for 1 h at RT with serially diluted rat sera and then for 1 h with digoxigenin (DIG)-coupled antigen diluted to 0.2 µg/mL (10∶1, DIG-Ara h 2) or 0.8 µg/mL (3.5∶1; DIG-HAra h 1, DIG-GAra h 1) in PBS-T containing 3% rabbit serum. After washing, plates were incubated with HRP-labeled sheep anti-DIG (1∶1000; Roche, Mannheim, Germany) before development for 20–30 min in the dark. Detection limits were calculated to an absorbance value of 0.1. Native Ara h 1 (NAra h 1) and Ara h 2 were coupled using a DIG protein-labeling kit from Roche whereas HAra h 1 and GAra h 1 were coupled using Chromalink digoxigenin one-shot antibody labeling kit (SoluLink, San Diego, CA, USA) as the kit from Roche was no longer available.

#### Sandwich ELISA for measurement of total IgE

Total IgE responses were measured for serum pools by coating plates overnight at 4°C with 0.5 µg/mL of mouse anti-rat IgE (HPMAB-123 HybriDomus, Cytotech) in carbonate buffer. After blocking of remaining active sites for 1 h at 37°C with PBS-T, plates were incubated for 1 h at RT with serially diluted rat sera (starting at 1∶20) and rat IgE standard (Rat Myeloma IgE, 02-9788, Zymed). After washing, plates were incubated with HRP-labeled mouse anti-rat IgE (1∶2000; MCA193P, AbD Serotec, Düsseldorf, Germany) for 1 h at RT before development for 20 min in the dark.

Concentrations of total IgE in serum samples were interpolated from the linear part of the standard curve for purified IgE (three-parameter analysis, KC4 version 2.7). The average concentration of at least two serum dilutions was used for final calculation.

### Rat Basophilic Leukemia (RBL) Assay

Sera were examined by RBL assay using RBL-2H3 cells (DSMZ, Braunschweig, Germany). Cells were cultured, harvested and plated in flat-bottomed cell culture microtitre plates (Nunc) for attachment as described in Kroghsbo *et al*. [35] (1.5×10^5^ cells/well). Attached cells were sensitized passively with 50 µL/well of serum pools from i.p. sensitization study (diluted 1∶2) or feeding studies (undiluted), then washed twice before incubation with 100 µL/well of ten-fold diluted purified antigen solutions (0.01–100 µg/mL) or four-fold diluted peanut extract solutions (0.06–1000 µg/mL) for cross-linking. After incubation for 1 h plates were centrifuged and 25 µL supernatant (*Specific release*) was transferred to a microtitre plate (Nunc). Enzymatic activity in supernatants was detected by hydrolysis of the substrate *p*-nitro-phenyl-*N*-acetyl β-D-glucosamimide (PNAG; N9376, Sigma, Saint Louis, MO, USA) by addition of 100 µL/well and incubation for 90 min at 37°C. Reaction was terminated by addition of 100 µL/well of 0.2 M glycine solution, pH 10.7 (G7126, Sigma). *β*-hexosaminidase release was quantitatively measured spectrophotometrically at 405 nm with a reference wavelength of 630 nm using a microplate reader (BioTek Instruments Inc.). Total release from remaining intact cells was measured for each well by addition of detergent (80 µL/well of 0.2% Triton X-100; X100, Sigma), incubated for 30 min before centrifugation and transfer of 25 µL supernatant (*Total release*) to a second microtitre plate.

For control of IgE-mediated degranulation (*Total IgE release*), serum-sensitized cells were stimulated with 1.25 µg mouse anti-rat IgE/mL (553914, BD Pharmingen, San Diego, CA, USA) found to be the optimal concentration for biological release in initial optimization studies. Negative controls (non-sensitized cells or cells sensitized with naïve sera stimulated with peanut extracts) were added to each plate to measure spontaneous release.

For each serum sample *β*-hexosaminidase release was calculated according to the following equation:
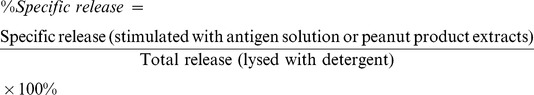



As total release was 30–40% for all serum pools and no statistically significant difference was found between groups, results are expressed as percent of maximum biological release:
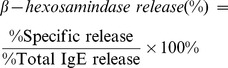



### Statistical Analysis

All statistical calculations on data were carried out using GraphPad Prism version 4.00 for Windows (GraphPad Software). ELISA results expressed as antibody titers were examined using non-parametric statistical analysis because data was not normally distributed for all experimental groups. Results from inhibition ELISAs were analyzed by one-way ANOVA followed by Tukey’s multiple comparison test. The Kruskal–Wallis test followed by Dunn’s multiple comparison test was used for comparison of more than three groups, RBL results were examined using a two-way ANOVA.

Differences between experimental groups were regarded as significant when *p* ≤ 0.05.

## Results

Dosing or blood drawing of animals did not result in any adverse events.

### Protein Profiles of Peanut Product Extracts Differ in Solubility of Peanut Allergens

Ara h 1 and Ara h 2 monomers content in extracts of roasted peanuts and peanut butter was estimated to be decreased 7.1–11.5 fold for Ara h 1 and 1.3–2.5 fold for Ara h 2/6 compared to the content in the blanched peanut extract reflecting formation of insoluble aggregates during roasting (Figure 1, Table 1).

**Table 1:** Composition of peanut extracts determined by densitometric analysis of lanes from SDS PAGE gel.

	Proteins %		
	Blanched PE*	Roasted PE	PB extract
**7S (Ara h 1)**	19	1.6	2.6
**11S (Ara h 3/4)**	57	86	85
**2S (Ara h 2/6)**	7.2	3.1	2.9

*PE: peanut extract, PB: peanut butter.

### Ara h 1 and Ara h 2 Induce More Specific IgE when Present in Roasted Peanuts Compared to Blanched Peanuts and Peanut Butter

**Figure 2:**
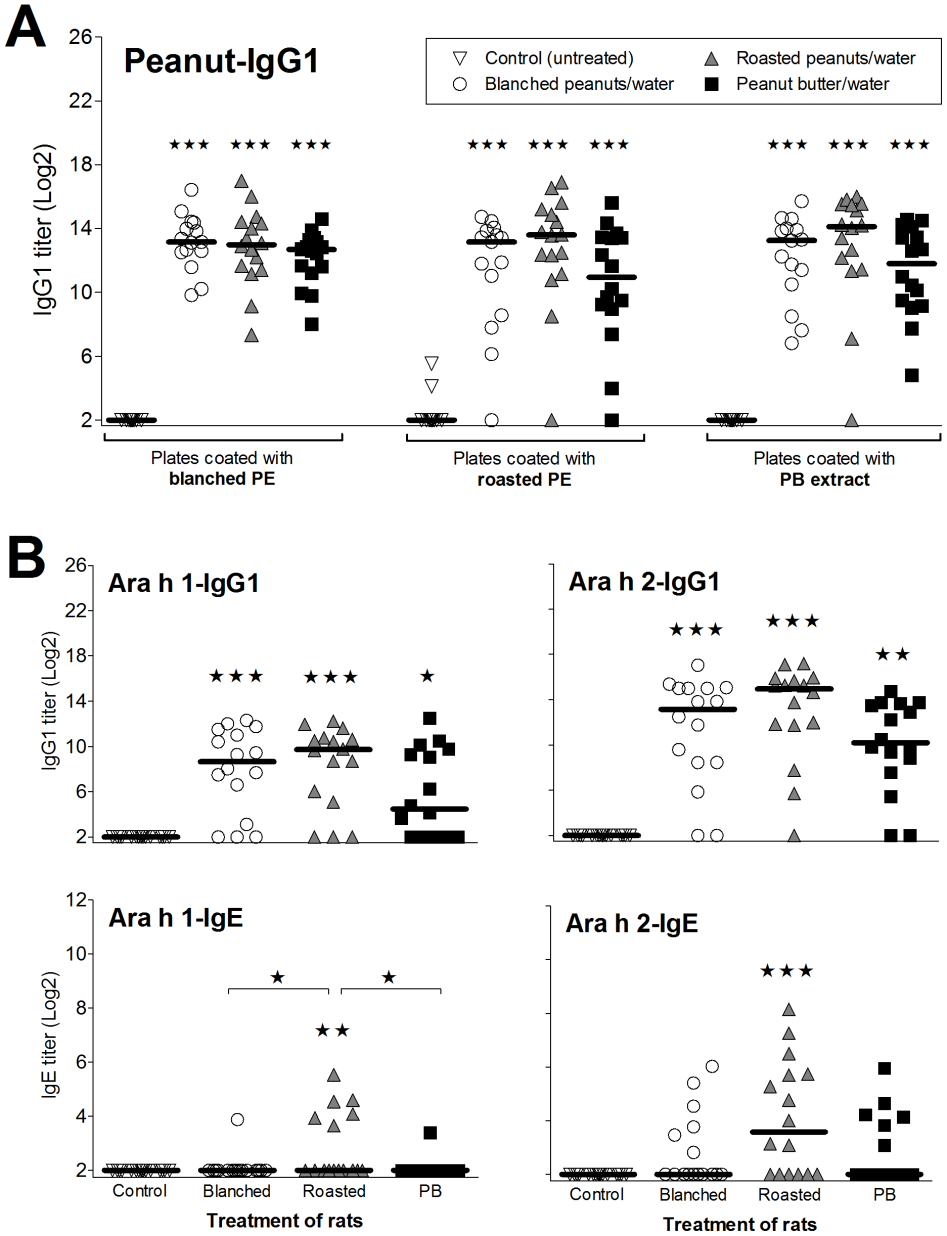
IgG1 and IgE response in sera from rats dosed orally with peanut products. Groups of BN rats were dosed for 42 days (day 1–42) with peanut-water mixtures prepared with blanched peanuts (open circles), roasted peanuts (grey triangles) or peanut butter (black squares) corresponding to approximately 2 mg Ara h 1 and 1 mg Ara h 2 per rat per day. Groups of untreated rats were included as controls (open triangles). Serum samples were obtained at sacrifice (day 49) and analyzed by ELISA. Sera were analyzed for specific IgG1 response against extracts of peanut-water mixtures (A) and against Ara h 1 and Ara h 2 purified from raw peanuts (B). Each symbol represents an animal. Horizontal bars indicate median values. Data were analyzed by non-parametrical test. Asterisks indicate statistically significant difference of a dosed group compared to the corresponding control group. Asterisks over a horizontal line indicate statistically significant difference between the two given groups. *: *p* ≤ 0.05; **: *p* ≤ 0.01; ***: *p* ≤ 0.001.

To examine whether processing and food matrix influence sensitization potential of peanut allergens, rats were dosed orally by gavage with water mixtures of blanched or roasted peanuts or peanut butter. As shown in Figure 2 all three peanut products induced a statistically significant IgG1 response against whole extracts (Figure 2A) and against Ara h 1 and Ara h 2, purified from raw peanuts (Figure 2B). Although not statistically significant, blanched and roasted peanuts had a tendency to induce more anti-Ara h 1 and anti-Ara h 2 IgG1 antibodies than peanut butter (Figure 2B). The Ara h 1- and Ara h 2-specific IgG2a response resembled the corresponding specific IgG1 response for all groups (data not shown) for which reason only results for specific IgG1 are shown.

Only oral dosing with roasted peanuts induced a statistically significant IgE response against Ara h 1 and Ara h 2 (Figure 2B). Specific IgE against peanut extracts was not assayed because coupling of peanut extracts to digoxigenin (DIG) most likely would result in ratios of coupled peanut proteins and peptides not resembling the actual ratios present in the extracts. Moreover specific IgE against extracts was not measured by direct ELISAs as the presence of a manifold higher concentration of IgG in the serum samples competes with IgE and dramatically lower assay sensitivity. No difference in total IgE was found between dosed groups or for the control group compared to dosed groups (data not shown).

### Extract from Roasted Peanuts is a Superior Elicitor in RBL Assay, but the Biological Functionality does not Reflect Specific IgE Titers

**Figure 3:**
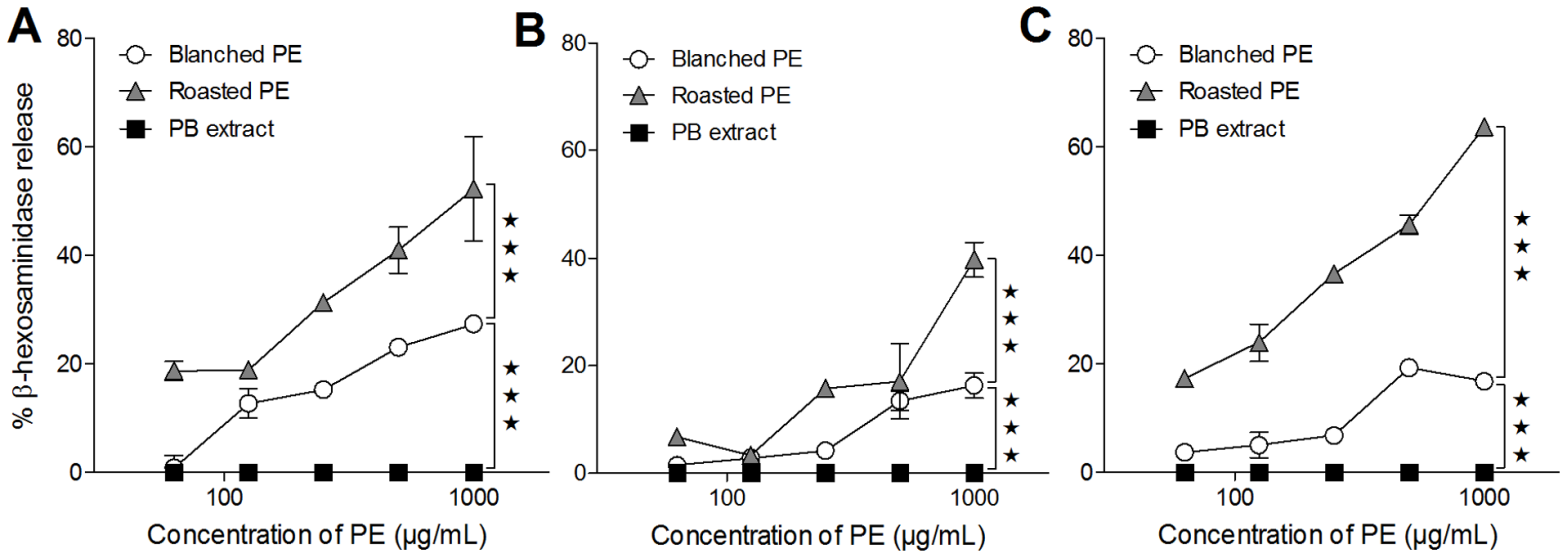
Allergen-specific degranulation of RBL cells sensitized with sera from rats dosed orally with peanut products. RBL cells were passively sensitized with serum pools (undiluted) from groups of BN rats dosed orally by gavage for 42 days with with blanched peanuts (**A**), roasted peanuts (**B**) and peanut butter (**C**) corresponding to approximately 2 mg Ara h 1 per rat per day. Peanut-water mixtures were prepared using blanched or roasted peanuts or peanut butter. For degranulation, cells were stimulated with dilutions of extracts of peanut-water mixtures (blanched PE, roasted PE or PB extract). Data are presented as percentage *β*–hexosaminidase release of total biological release induced by stimulation with 125 ng/well of anti-rat IgE. Symbols represent mean values ± SD for each serum pool. It was not possible to detect any degranulation of RBL cells when sensitized cells were stimulated with native or processed Ara h 1. The experiment was performed twice. Data were analyzed by two-way ANOVA. Statistical difference between extracts to induce allergen-specific degranulation is only indicated for least significant lines; ***: *p* ≤ 0.001. RBL: rat basophilic leukemia, PE: peanut extract, PB: peanut butter.

Biological functionality of the specific IgE responses, i.e. whether it may induce elicitation of an allergic reaction, was analyzed using RBL assay by sensitizing cells with serum pools from each group of rats before stimulating with peanut product extracts (Figure 3). Regardless of the peanut product used for sensitization by gavage, extract from roasted peanut induced a statistically significant higher degranulation of sera-sensitized RBL cells compared to blanched peanut extract whereas no elicitation was observed for stimulation with peanut butter extract. While ELISA analysis showed the highest Ara h 1- and Ara h 2-specific IgE response induced by oral dosing with roasted peanuts (Figure 2B) examination of the same sera by RBL assay using extracts for stimulation showed a higher biological activity of specific IgE when rats were dosed with blanched peanuts or peanut butter compared to dosing with roasted peanuts (Figure 3).

It was not possible to detect any significant degranulation of RBL cells when sensitized cells were stimulated with purified native or processed Ara h 1 or purified native Ara h 2 (data not shown).

### Heating and Heat Glycation Change the Immune Response to Ara h 1

**Figure 4:**
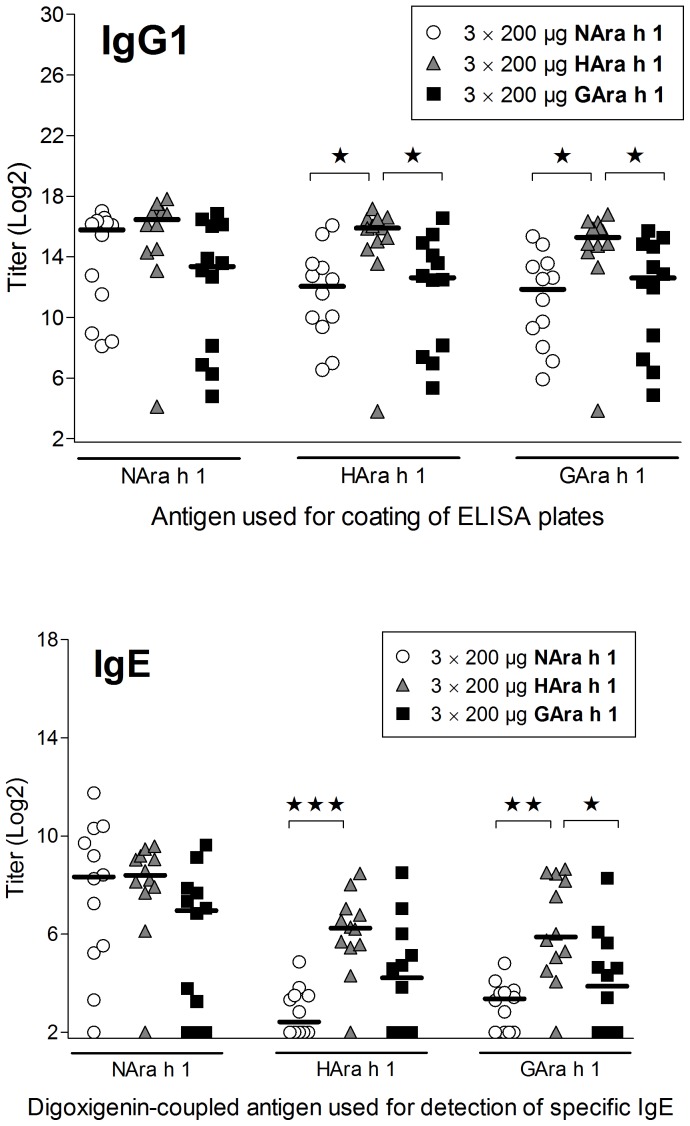
Specific IgG1 and IgE response in sera from rats immunized i.p. with native or processed Ara h 1. Groups of BN rats were immunized on day 0, 14 and 28 with 200 µg of NAra h 1 (open circles), HAra h 1 (grey triangles) or GAra h 1 (black squares). Serum samples were obtained at sacrifice (day 35) and analyzed by ELISA for specific IgG1 and IgE against native and processed Ara h 1. No allergen-specific IgG1 or IgE was detected for control animals (data not shown). Each symbol represents an animal. Horizontal bars indicate median values. Data were analyzed by non-parametrical test. Asterisks over a horizontal line indicate statistically significant difference between the two given groups. *: *p* ≤ 0.05; **: *p* ≤ 0.01; ***: *p* ≤ 0.001.

**Figure 5:**
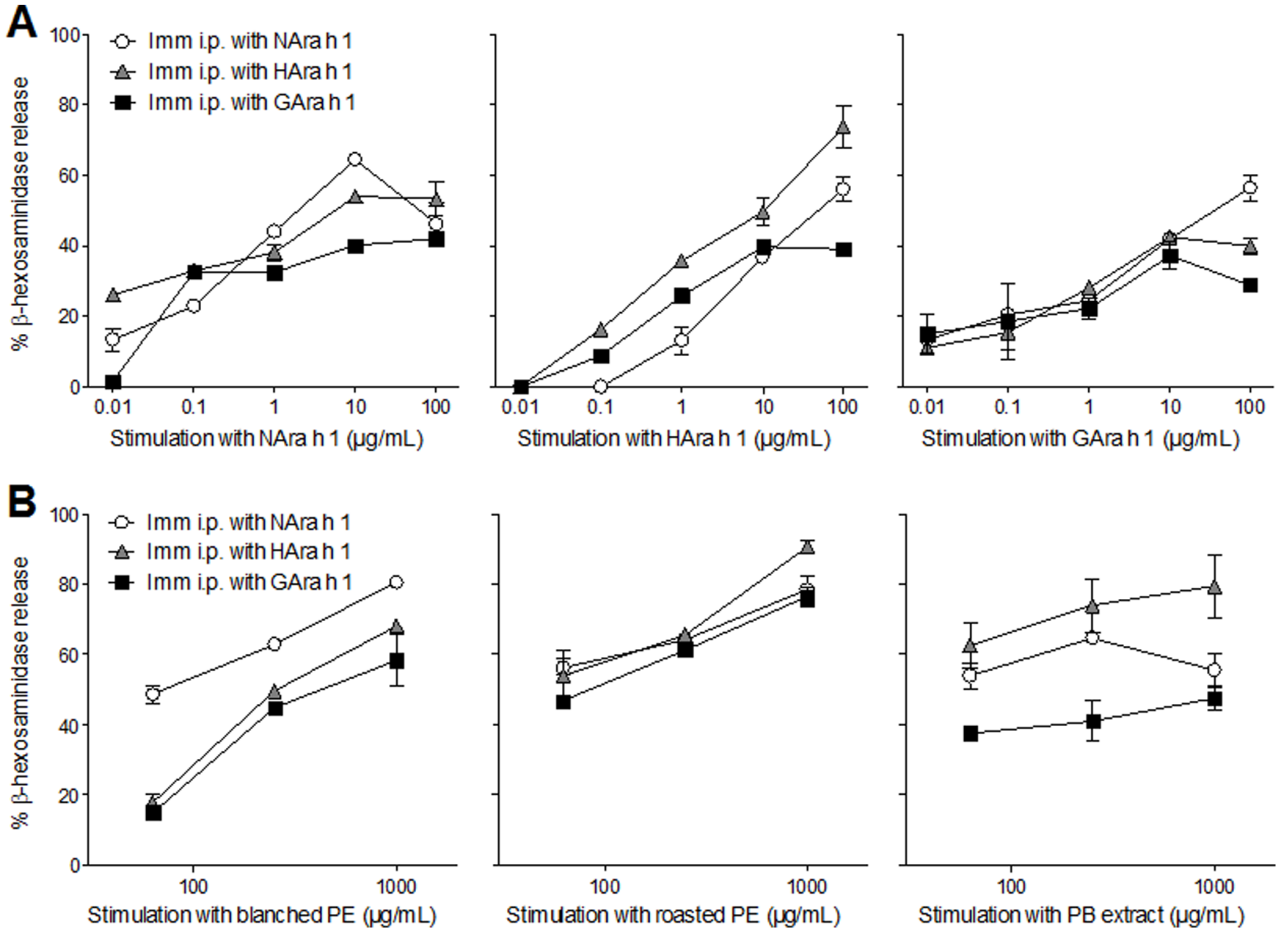
Allergen-specific degranulation of RBL cells sensitized with sera from i.p. study with Ara h 1. RBL cells were passively sensitized with serum pools (diluted 1∶2) from groups immunized i.p. three times with 200 µg of NAra h 1 (open circles), HAra h 1 (grey triangles) or GAra h 1 (black squares). For degranulation cells were stimulated with dilutions of native or processed Ara h 1 (A) or extracts of peanut-water mixtures (B). Data are presented as percentage *β*–hexosaminidase release of total biological release induced by stimulation with 125 ng/well of anti-rat IgE. Symbols represent mean values ± SD for each serum pool. RBL: rat basophilic leukemia, Imm: immunization, PE: peanut extract, PB: peanut butter.

Analysis of sera from rats immunized i.p. with native (NAra h 1) or processed Ara h 1 (HAra h 1 or GAra h 1) by ELISA showed that all three Ara h 1 products had sensitizing potential and induced a statistically significant specific IgG1 (Figure 4) and IgG2a (data not shown) response. No significant difference in IgG1 response to NAra h 1 was seen between groups. Animals immunized with HAra h 1 had a statistically significant higher IgG1 response against HAra h 1 and GAra h 1 compared to animals sensitized with NAra h 1 or GAra h 1. The same pattern was found for specific IgE responses (Figure 4) suggesting that new epitopes are formed or disclosed by heating without glucose. Because of the nature of the IgE ELISA where process modification may influence the DIG coupling, we have not tried to compare IgE ELISA results between N-, H- or GAra h 1. It may be more relevant to compare the biologic activity of IgE responses i.e. the degranulation of RBL cells sensitized with IgE against N-, H- or GAra h 1 and degranulated with the respective allergen. RBL results showed that sensitization to NAra h 1 and HAra h 1 induced comparable functional IgE responses at high stimulation concentrations whereas GAra h 1 induced a slightly lower response (Figure 5A). GAra h 1 seemed the least efficient in degranulating cells irrespective of IgE specificity. HAra h 1 was found to be the most efficient degranulator of RBL cells sensitized with IgE against HAra h 1, whereas IgE induced by NAra h 1 reacted equally well with native and processed Ara h 1 (Figure 5A). This is in accordance with the ELISA results where IgE titers to NAra h 1 were comparable, whereas the IgE titer to HAra h 1 was highest in animals sensitized with HAra h 1.

Taking account of the difference in Ara h 1 content of the extracts, extracts from the roasted products are overall more efficient elicitors of degranulation irrespective of the specificity of the IgE (Figure 5B).

### IgG1-binding Capacity of Sera Reflects whether Animals are Sensitized to Native or Processed Ara h 1 or Dosed with Blanched or Roasted Peanut Products

**Figure 6:**
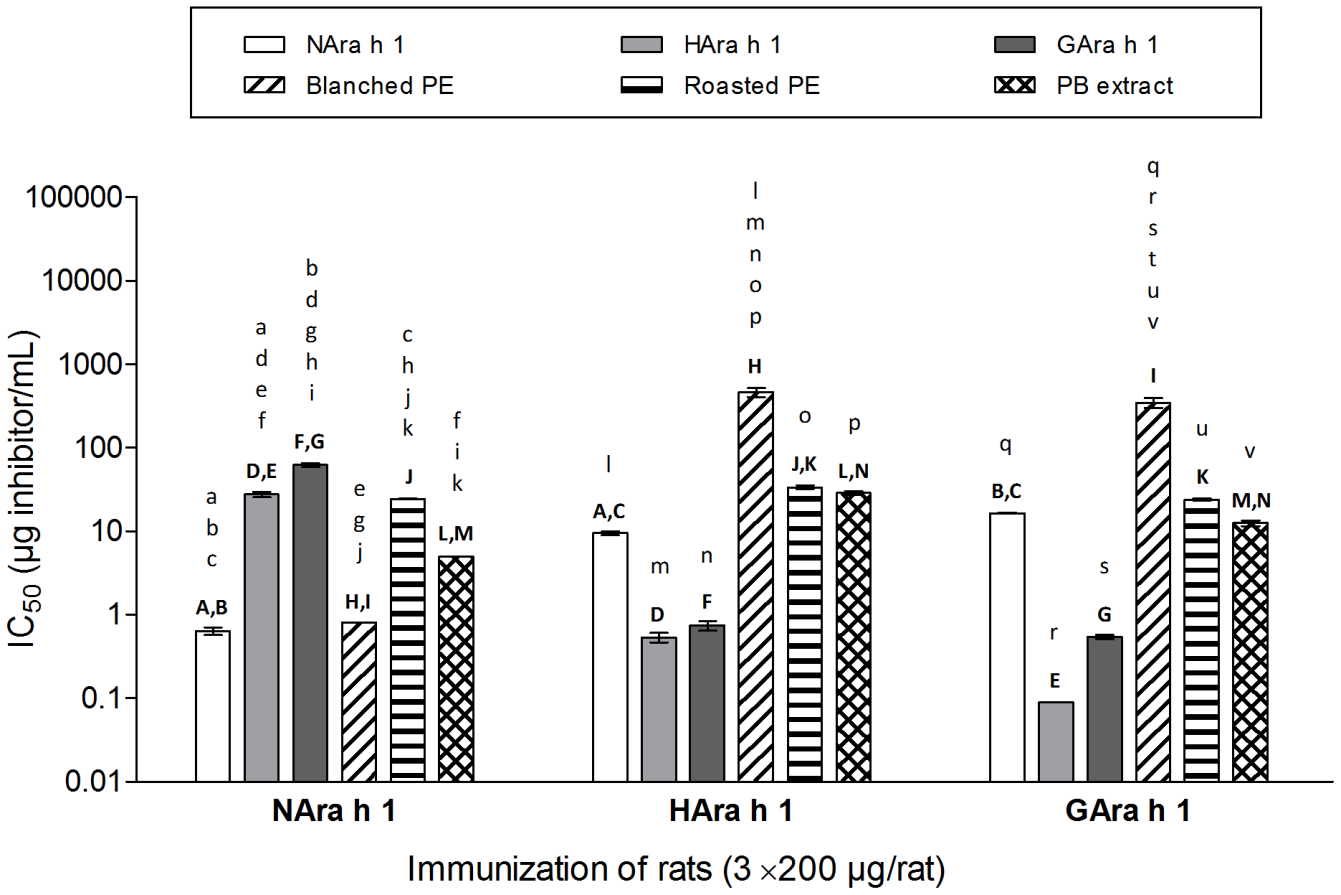
IgG1-binding capacity of sera from i.p. study with native and processed Ara h 1. Groups of rats were dosed i.p. on day 0, 14 and 28 with 200 µg of NAra h 1, HAra h 1 or GAra h 1. Serum pools obtained at sacrifice were preincubated with tenfold dilutions of native or processed Ara h 1 (0.0001 µg/mL–100 µg/mL) or peanut product extracts (0.001 µg/mL–1000 µg/mL) before measurement of IgG1-binding capacity by addition to ELISA plates coated with the antigen (N-, H- or GAra h 1) the group was sensitized against. Each bar represents IC_50_ mean values ± SD for a serum pool for each group of rats. Mean values were calculated as average of three measurements (each representing triplicate pipettings of the same serum/inhibitor mix) performed within one month. Data were analyzed by one-way ANOVA followed by Tukey’s multiple comparison test. Statistically significant difference (*p* ≤ 0.05) is shown by letters where two groups with the same letter are significant different (lower case letters: intragroup analysis, upper case letters: intergroup analysis). PE: peanut extract, PB: peanut butter.

Results obtained by inhibition ELISAs showed that processed Ara h 1 (HAra h 1 and GAra h 1) were better inhibitors of IgG1-binding using sera from animals immunized with processed Ara h 1 compared to NAra h 1 (Figure 6). Also extracts of roasted peanuts and peanut butter showed higher IgG1-binding capacity than blanched peanut extract for groups immunized with processed Ara h 1 even though the Ara h 1 content of these extracts are much lower than that of the blanched peanut extract. IgG1-binding capacities of NAra h 1 and blanched peanut extracts were higher in rats sensitized with NAra h 1 (Figure 6).

## Discussion

It has been suggested that the worldwide variation in peanut allergy prevalence may be caused by differences in peanut processing rather than being caused by differences in consumption. In China where peanuts are used as stable foods and boiled or fried, the peanut prevalence seems to be lower than in countries where peanuts are being consumed roasted as snack or in peanut butter [13], [24]. This has been supported by studies showing that roasting increases IgE-binding [11], [12] and that boiling or frying [13] reduces IgE-binding. Mondoulet *et al*. [14] was not able to confirm that roasting increases IgE-binding when analyzing whole peanut protein extracts but found that boiling released low-molecular weight allergens into the boiling water, explaining the lower IgE-binding of boiled peanuts. Recently, Blanc *et al*. [17] showed that IgE-binding capacity of roasted Ara h 1 was similar to native Ara h 1 whereas boiling of purified Ara h 1 reduced IgE-binding irrespective of the presence or absence of glucose during heating. The authors suggest that the difference in IgE-binding may be explained by formation of distinct morphological aggregates; roasted Ara h 1 comprised of compact globular aggregates with a higher content of native-like *β*-sheet structures compared to the more ‘rod-like’ branched aggregates induced by boiling.

We have used the well described BN rat as model for sensitization as it is very difficult to study sensitization in humans, because it is nearly impossible to know the exact exposure. We study the functionality of the response using an *in vitro* assay rather than burden the animals by challenge. This would also include the use of adjuvants in the sensitization process, which we avoid.

Only few studies have examined the sensitization potential of whole foods and to our knowledge the results presented herein are the first comparing how processing affects sensitization potential of whole foods and also the first study to examine sensitization potential of processed Ara h 1. As peanut processing decreases protein solubility [[37], [38]; dosing with food extract (comprising of only soluble proteins) may bias result interpretation. On the other hand, at the moment the only way to perform *in vitro* analysis of the functionality of specific IgE and determination of specific IgE-binding is by the use of extracted soluble proteins.

Allergenicity in the terms of IgE-binding and mediator release assays have been used for examination of how different processing methods affect the allergenicity of peanut allergens. Nevertheless, these assays only reveal something about elicitation and not sensitization potential of food allergens.

We found that oral dosing of rats with roasted peanuts induced significant anti-Ara h 1 and anti–Ara h 2 IgE responses when measured by ELISA. In rats dosed with blanched peanuts or peanut butter a significant number of rats (5 out of 16 rats per group) developed IgE to Ara h 2, but not to Ara h 1 (1 out of 16). This difference in IgE titer to Ara h 1 and 2 is not reflected in the results of the RBL assay showing that sera from rats dosed with roasted peanuts induced a lower functional IgE response compared to sera from rats dosed with blanched peanuts and peanut butter when degranulation was induced by stimulation with the roasted peanut extract. In a previous study [35], we showed that IgE titer may not always predict IgE functionality in BN rats which is supported by the findings that heated Ara h 1 possessed increased capacity to elicit mediator release although IgE-binding was reduced [16], [17]. Weighting functionality based on whole peanut extracts higher than IgE titer against Ara h 1 and 2, our results show that roasted peanuts as such or in peanut butter are not more efficient to sensitize compared to blanched peanuts and that blanched peanuts and peanut butter (produced from roasted peanuts) are equally good as sensitizers. However, these results may be biased as it cannot be excluded that allergen(s) efficient for degranulation was altered through processing or not extracted from the peanut matrices.

Roasting of peanuts results in a significant decrease in protein solubility that may influence digestibility. How this exactly influences the ability to sensitize is difficult to predict as the solubility increases dramatically at pH 2 [37]. Ara h 2 from roasted peanuts has been found to possess increased trypsin inhibitory activity suggesting its role as a protector of other peanut proteins to proteolytic digestion [38]. We have previously shown that digested Ara h 1 has both sensitizing and eliciting properties [39]. To our knowledge, no previous study of peanut and peanut extract has compared sensitization to different peanut products. Sensitization potential of ground (presumably roasted) peanuts [30] and peanut extracts (probably from raw peanuts) with and without fat [31] have been studied in mice using oral dosing with cholera toxin as mucosal adjuvant showing that both peanut products were able to induce specific IgE. The fat content did not influence sensitization [31]. In summary, based on available information, we are not able to explain why oral sensitization to roasted peanuts induce a less functional IgE response than peanut butter and blanched peanuts.

Furthermore, the RBL assay also showed that irrespective of the peanut product used for sensitization, extract from roasted peanuts is a far better elicitor than extract from blanched peanuts and peanut butter. This may be explained by the fact that roasting increases aggregation of proteins [25], [37] and that aggregates may be better elicitors of basophil degranulation.

Extracting proteins from processed peanuts may be difficult as the protein solubility is decreased by processing [25], [37], [40]–[42]. In this study the concentration of proteins extracted from blanched peanuts was nearly twofold higher compared to roasted peanuts and peanut butter. The extracts were therefore adjusted with respect to protein concentration [37].

The Ara h 1 and Ara h 2 content in extracts from roasted peanuts and peanut butter was lower than in the blanched peanut extract indicating that roasting decreases solubility of these proteins, in particularly Ara h 1. As total protein concentration was adjusted and the Ara h 1 and Ara h 2 content of processed peanuts was lower, the relative concentration of the other allergens has been higher in the roasted peanut extract compared to the blanched peanut extract.

In this way, the more efficient elicitation mediated by the roasted peanut extract may be a true difference but could also reflect dissimilarity in protein distribution as a consequence of difference in solubility. Ara h 2/6 has been reported to be much more potent elicitors of basophil degranulation compared to Ara h 1. Also Ara h 3 has been reported to be a more potent allergen than Ara h 1 [16], [43]. It is not possible to elucidate how the decrease in Ara h 1 and 2 and increase in Ara h 3/4 may have influenced the RBL result. Unfortunately there are no clinical challenge studies in peanut allergic patients comparing raw, blanched or roasted peanuts so the possible clinical implication of the superior elicitation caused by roasted peanut extract remains unknown.

Examination of sensitization capacity of purified proteins can be difficult or in some cases impossible to perform in an oral model because of the amount of protein required. For this reason we chose to study heated and glycated Ara h 1 using i.p. immunization. The disadvantage of this model is that it bypasses the proteolytic environment of the gastrointestinal tract. As heating and glycation of Ara h 1 did not change *in vitro* digestibility (unpublished data) we found it justified comparing the sensitizing potential of native and process-modified Ara h 1 using the i.p. model.

Processing did not increase or decrease the sensitizing capacity of purified Ara h 1. However ELISA results showed differences in reactivity especially between IgG1 antibodies to NAra h 1 and HAra h 1 suggesting that new epitopes are formed or disclosed by heating and to a lesser degree by glycation. This is also reflected in the inhibition ELISA results where NAra h 1 is a better inhibitor for sera raised against NAra h 1 and HAra h 1 is a better inhibitor for sera raised against HAra h 1 or GAra h 1. The same pattern was seen when extracts were used as inhibitor. Here blanched peanut extract was a very week inhibitor for sera raised against HAra h 1 or GAra h 1 although the Ara h 1 content is much higher than in the extracts from roasted peanut products. Together, the results obtained by RBL assay and inhibition ELISA indicate that the process modification of purified Ara h 1 caused by heating with or without glucose actually resembles what goes on in the peanut during the roasting procedure. Extract from roasted peanuts is a better elicitor and inhibitor of sera against HAra h 1 and GAra h 1 compared to extract from blanched peanuts especially when the higher Ara h 1 content in the blanched peanut extract is taken into account. This is in accordance with the finding that Ara h 1 purified from (dry-)roasted peanuts has similar IgE-binding capacity as native Ara h 1 despite being denatured and highly aggregated while boiling of Ara h 1 reduced IgE-binding capacity [17]. In contrary, other studies have shown increased IgE-binding of Ara h 1 after processing [12], [14].

In addition, it seems that blanching for up to 15 min at 120–130°C induces much less protein change compared to roasting, as proteins are readily extracted and the distribution of allergens resembles the allergen content of peanuts reported in the literature [43]. Extract from blanched peanuts is a better elicitor and inhibitor of sera against NAra h 1 (from raw peanut) compared to extract from roasted peanuts even when the difference in Ara h 1 content is taken into account.

We have shown that IgE from animals sensitized with heated Ara h 1 has higher binding capacity to heated Ara h 1 compared to Ara h 1 from blanched peanuts. It is worth noting that studies comparing IgE-binding capacity of heat modified peanut allergens or products have used sera from patients that most probably have been sensitized to roasted peanuts either as such or in peanut butter [12]–[14]. In the light of our findings, the differences in IgE-binding of processed peanuts or processed peanut allergens using human sera may also be influenced by the initial sensitization.

In conclusion our results show that peanut products may sensitize BN rats and induce a functional specific IgE response when dosed orally without adjuvant. Roasted peanuts, either as such or as peanut butter, induced higher IgE anti-Ara h 1 and anti-Ara h 2 titers than blanched peanuts but these IgE showed low functionality in our cellular test. This is supported by the finding that process-modified Ara h 1 has a similar sensitizing capacity as native Ara h 1. On the other hand we found that irrespective of the peanut product used for sensitization, extract from roasted peanuts is a better elicitor than extract from blanched peanuts.

## References

[1] Breiteneder H, Mills ENC (2005) Molecular properties of food allergens. J Allergy Clin Immunol 115: 14–23. 15637541 10.1016/j.jaci.2004.10.022

[2] Mills ENC, Sancho AI, Rigby NM, Jenkins JA, Mackie AR (2009) Impact of food processing on the structure and allergenic properties of food allergens. Mol Nutr Res 53: 963–969. 10.1002/mnfr.200800236 19603402

[3] Nowak-Wegrzyn A, Fiocchi A (2009) Rare, medium, or well done? The effect of heating and food matrix on food protein allergenicity. Curr Opin Allergy Clin Immunol 9: 234–237. 19444093 10.1097/ACI.0b013e32832b88e7

[4] Grimshaw KEC, King RM, Nordlee JA, Hefle SL, Warner JO, et al (2003) Presentation of allergen in different food preparations affects the nature of the allergic reaction – a case series. Clin Exp Allergy 33: 1581–1585. 14616872 10.1046/j.1365-2222.2003.01795.x

[5] Paschke A (2009) Aspects of food processing and its effect on allergen structure. Mol Nutr Food Res 53: 959–962. 19557818 10.1002/mnfr.200800187

[6] Mills ENC, Mackie AR (2008) The impact of processing on allergenicity of food. Curr Opin Allergy Clin Immunol 8: 249–253. 18560301 10.1097/ACI.0b013e3282ffb123

[7] Ladics GS (2009) Food Processing and Allergenicity. Mol Nutr Food Res 53: 945. 19639611 10.1002/mnfr.200990025

[8] Sathe SK, Sharma GM (2009) Effects of food processing on food allergens. Mol Food Res 53: 970–978. 10.1002/mnfr.200800194 19603400

[9] Davis PJ, Williams SC (1998) Protein modification by thermal processing. Allergy 53: 102–105. 10.1111/j.1398-9995.1998.tb04975.x 9826012

[10] Taheri-Kafrani A, Gaudin J-C, Rabesona H, Nioi C, Agarwal D, et al (2009) Effects of heating and glycation of β-lactoglobulin on its recognition by IgE of sera from cow milk allergy patients. J Agric Food Chem 57: 4974–4982. 19489627 10.1021/jf804038t

[11] Chung S-Y, Champagne ET (2001) Association of end-product adducts with increased IgE binding of roasted peanuts. J Agric Food Chem 49: 3911–3916. 11513688 10.1021/jf001186o

[12] Maleki MJ, Chung S-Y, Champagne ET, Raufman J-P (2000) The effects of roasting on the allergenic properties of peanut proteins. J Allergy Clin Immunol 106: 763–768. 11031348 10.1067/mai.2000.109620

[13] Beyer K, Morrow E, Li X-M, Bardina L, Bannon GA, et al (2001) Effects of cooking methods on peanut allergenicity. J Allergy Clin Immunol 107: 1077–1081. 11398088 10.1067/mai.2001.115480

[14] Mondoulet L, Paty E, Drumare MF, Ah-Leung S, Scheinmann P, et al (2005) Influence of thermal processing on the allergenicity of peanut proteins. J Agric Food Chem 53: 4547–4553. 15913323 10.1021/jf050091p

[15] Vissers YM, Blanc F, Skov PS, Johnson PE, Rigby NM, et al (2011) Effect of Heating and Glycation on the Allergenicity of 2S Albumins (Ara h 2/6) from peanut. PLoS ONE 6(8): e23998 doi:10.1371/journal.pone.0023998 21901150 10.1371/journal.pone.0023998 PMC3162016

[16] Vissers YM, Iwan M, Adel-Patient K, Stahl Skov P, Rigby NM, et al (2011) Effect of roasting on the allergenicity of major peanut allergens Ara h 1 and Ara h 2/6: the necessity of degranulation assays. Clin Exp Allergy 41: 1631–1642. 21801247 10.1111/j.1365-2222.2011.03830.x

[17] Blanc F, Vissers YM, Adel-Patient K, Rigby NM, Mackie AR, et al (2011) Boiling peanut Ara h 1 results in the formation of aggregates with reduced allergenicity. Mol Nutr Food Res 55: 1887–1894. 22086730 10.1002/mnfr.201100251

[18] Nakamura S, Suzuki Y, Ishikawa E, Yakushi T, Jing H, et al (2008) Reduction of in vitro allergenicity of buckwheat Fag e 1 through the Maillard-type glycosylation with polysaccharides. Food Chem 109: 538–545.

[19] Nakamura A, Watanabe K, Ojima T, Ahn D-H, Saeki H (2005) Effects of Maillard reaction on allergenicity of scallop tropomyosin. J Agric Food Chem 53: 7559–7564. 16159186 10.1021/jf0502045

[20] Nakamura A, Sasaki F, Watanabe K, Ojima T, Ahn D-H, et al (2006) Changes in allergenicity and digestibility of squid tropomyosin during the Maillard reaction with ribose. J Agric Food Chem 54: 9529–9534. 17147442 10.1021/jf061070d

[21] Gruber P, Vieths S, Wangorsch A, Nerkamp J Hofmann T (2004) Maillard reaction and enzymatic browning affect the allergen from cherry (Prunus avium). J Agric Food Chem 52: 4002–4007. 15186129 10.1021/jf035458+

[22] Sancho AI, Rigby NM, Zuidmeer L, Asero R, Mistrello G, et al (2005) The effect of thermal processing on the IgE reactivity of the non-specific lipid transfer protein from apple, Mal d 3. Allergy 60: 1262–1268. 16134992 10.1111/j.1398-9995.2005.00876.x

[23] Iwan M, Vissers YM, Fiedorowicz E, Kostyra H, Kostyra E, et al (2011) Impact of Maillard reaction on immunoreactivity and allergenicity of the hazelnut allergen Cor a 11. J Agric Food Chem 59: 7163–7171. 21563837 10.1021/jf2007375

[24] Boulay A, Houghton J, Gancheva V, Sterk Y, Strada A, et al (2008) A EuroPrevall review of factors affecting incidence of peanut allergy: priorities for research and policy. Allergy 63: 797–809. 18588545 10.1111/j.1398-9995.2008.01776.x

[25] Koppelman SJ, Bruijnzeel-Koomen CAFM, Hessing M, de Jongh HHJ (1999) Heat-induced conformational changes of Ara h 1, a major peanut allergen, do not affect its allergenic properties. J Biol Chem 274: 4770–4777. 9988715 10.1074/jbc.274.8.4770

[26] Maleki SJ, Hurlburt BK (2004) Structural and functional alterations in major peanut allergens caused by thermal processing. J AOAC Int 87: 1475–1479. 15675461

[27] Lehmann K, Schweimer K, Reese G, Randow S, Suhr M, et al (2006) Structure and stability of 2S albumin-type peanut allergens: implications for the severity of peanut allergic reactions. Biochem J 395: 463–472. 16372900 10.1042/BJ20051728 PMC1462689

[28] Chung S-Y, Butts CL, Maleki SJ, Champagne ET (2003) Linking peanut allergenicity to the processes of maturation, curing, and roasting. J Agric Food Chem 51: 4273–4277. 12848497 10.1021/jf021212d

[29] Gruber P, Becker W-M, Hofmann T (2005) Influence of the Maillard reaction on the allergenicity of rAra h 2, a recombinant major allergen from peanut (Arachis hypogaea), its major epitopes, and peanut agglutinin. J Agric Food Chem 53: 2289–2296. 15769170 10.1021/jf048398w

[30] Li XM, Serebrisky D, Lee SY, Huang CK, Bardina L, et al (2000) A murine model of peanut anaphylaxis: T- and B-cell responses to a major peanut allergen mimic human responses. J Allergy Clin Immunol 106: 150–158. 10887318 10.1067/mai.2000.107395

[31] van Wijk F, Nierkens S, Hassing I, Feijen M, Koppelman SJ, et al (2005) The effect of the food matrix on in vivo immune responses to purified peanut allergens. Toxicol Sci 86: 333–341. 15858220 10.1093/toxsci/kfi187

[32] Marsh J, Rigby N, Wellner K, Reese G, Knulst A, et al (2008) Purification and characterisation of a panel of peanut allergens suitable for use in allergy diagnosis. Mol Nutr Food Res 52: 272–285. 10.1002/mnfr.200700524 18727014

[33] Johnson PE, Van der Plancken I, Balasa A, Husband FA, Grauwet T, et al (2010) High pressure, thermal and pulsed electric-field-induced structural changes in selected food allergens. Mol Nutr Food Res 54: 1701–1710. 20568235 10.1002/mnfr.201000006

[34] Barkholt V, Jensen AL (1989) Amino acid analysis: Determination of cysteine plus half-cysteine in proteins after hydrochloric acid hydrolysis with a disulfide compound as additive. Anal Biochem 177: 318–322. 2729552 10.1016/0003-2697(89)90059-6

[35] Kroghsbo S, Bøgh KL, Rigby NM, Mills ENC, Rogers A, et al (2011) Sensitization with 7S globulins from peanut, hazelnut, soy or pea induces IgE with different biological activities which are modified by soy tolerance. Int Arch Allergy Immunol 155: 212–224. 21282960 10.1159/000321200

[36] Kroghsbo S, Madsen C, Poulsen M, Schrøder M, Kvist PH, et al (2008) Immunotoxicological studies of genetically modified rice expressing PHA-E lectin or Bt toxin in Wistar rats. Toxicology 245: 24–34. 18215453 10.1016/j.tox.2007.12.005

[37] Schmitt DA, Nesbit JB, Hurlburt BK, Cheng H, Maleki SJ (2010) Processing can alter the properties of peanut extract preparations. J Agric Food Chem 58: 1138–1143. 20028112 10.1021/jf902694j

[38] Maleki SJ, Viguez O, Jacks T, Dodo H, Champagne ET, et al (2003) The major peanut allergen Ara h 2, functions as a trypsin inhibitor, and roasting enhances this function. J Allergy Clin Immunol 112: 190–195. 12847498 10.1067/mai.2003.1551

[39] Bøgh KL, Kroghsbo S, Dahl L, Rigby NM, Barkholt V, et al (2009) Digested Ara h 1 has sensitizing capacity in Brown Norway rats. Clin Exp Allergy 39: 1611–1621. 19689460 10.1111/j.1365-2222.2009.03333.x

[40] Dyer S, Mack L, Cheng H, Maleki SJ (2008) Structural, chemical and immunogenic effects of roasting on Ara h 3, a major peanut allergen. J Allergy Clin Immunol 121: S183.

[41] Westphal CD, Pereira MR, Raybourne RB, Williams KM (2004) Evaluation of extraction buffers using the current approach of detecting multiple allergenic and nonallergenic proteins in food. J AOAC Int 87: 1458–1465. 15675459

[42] Poms RE, Capelletti C, Anklam E (2004) Effect of roasting history and buffer composition on peanut protein extraction efficiency. Mol Nutr Food Res 48: 459–464. 15508181 10.1002/mnfr.200400052

[43] Blanc F, Adel-Patient K, Drumare MF, Paty E, Wal JM, et al (2009) Capacity of purified peanut allergens to induce degranulation in a functional in vitro assay: Ara h 2 and Ara h 6 are the most efficient elicitors. Clin Exp Allergy 39: 1277–1285. 19538351 10.1111/j.1365-2222.2009.03294.x

